# Magnetic Resonance Imaging and Histopathologic Findings From a Standard Poodle With Neonatal Encephalopathy With Seizures

**DOI:** 10.3389/fvets.2020.578936

**Published:** 2020-11-10

**Authors:** Yoshihiko Yu, Daisuke Hasegawa, James K. Chambers, Kazuhiro Kojima, Rikako Asada, Gary S. Johnson, Kazuyuki Uchida

**Affiliations:** ^1^Laboratory of Veterinary Radiology, Nippon Veterinary and Life Science University, Tokyo, Japan; ^2^The Research Center for Animal Life Science, Nippon Veterinary and Life Science University, Tokyo, Japan; ^3^Laboratory of Veterinary Pathology, Graduate School of Agricultural and Life Sciences, The University of Tokyo, Tokyo, Japan; ^4^Department of Veterinary Pathobiology, University of Missouri, Columbia, MO, United States

**Keywords:** *ATF2*, activating transcription factor 2, canine, dog, EEG, epilepsy, MRI

## Abstract

Neonatal encephalopathy with seizures (NEwS) is an epileptic encephalopathy with an autosomal recessive inheritance pattern found in Standard Poodle puppies. The causal genetic variant for NEwS has been identified as a homozygous missense mutation in *ATF2* (c.152T>G, p.Met51Arg), and a pathological cerebellar change has been reported. Magnetic resonance imaging showed reduced whole-brain size, dilated ventricles, developmental abnormalities of the white matter of the cerebrum, white matter signal abnormalities in the occipital lobe, and abnormal morphology of the cerebellum. Histopathology included previously unrecognized irregular neuronal migration in the subventricular zone around the lateral ventricles in the frontal lobe and white matter rarefaction especially at the level of the occipital lobe in the cerebrum in addition to the cerebellar cortical dysplasia that has been previously described. The findings of this case may highlight the critical role of *ATF2* in neurodevelopmental processes in the canine brain.

## Background

Neonatal encephalopathy with seizures (NEwS; OMIA: 001471-9615), an autosomal recessive disease reported in Standard Poodle puppies, is caused by a homozygous missense mutation in the *activating transcription factor 2* (*ATF2*) gene (c.152T>G, p.Met51Arg) (1). In the previous report, ~36% of 1,038 Standard Poodles genotyped were found to be heterozygous carriers (1). *ATF2* encodes activating transcription factor 2 (ATF2) that regulates the transcription of various genes associated with anti-apoptosis, cell growth, and the DNA damage response. A previous study reported clinical information, including electroencephalography (EEG) findings, and cerebellar pathology (1). Nonetheless, the current report expands these findings with magnetic resonance imaging (MRI) and previously unrecognized histopathologic lesions. Herein we report the 3-Tesla MRI and detailed histopathological findings of NEwS in a Standard Poodle puppy in addition to the other clinical data including EEG findings.

## Case Presentation

A 64-days-old, 1.9 kg, intact female Standard Poodle dog was presented to the Laboratory of Veterinary Radiology, Nippon Veterinary and Life Science University (Tokyo, Japan), with a history of ataxia, generalized convulsive seizures, and weakness. This puppy and the six siblings were born alive at the owner's home, while the other sibling was stillborn. The affected puppy was almost the same size as others at birth, however the abnormalities were apparent by 3 days after birth. The puppy was too weak to suckle without owner's assistance and showed reduced growth compared to the siblings. At the second week after birth, ataxic gait became apparent. Additionally, after the third week of age, the puppy could no longer suckle and forced feeding was carried out by the owner. At that time the puppy did not interact with the siblings and dam, and showed dull responses to external stimuli.

This puppy developed generalized convulsive seizures (Supplementary Video 1) at 19 days of age. The seizures occurred almost every day while there were some days when seizures were not recognized. Seizures usually lasted 2–3 min, but some lasted more than 5 min and/or were clustered. Sometimes, vocalization was seen during seizures. Although prodrome was not evident, postictal phenomena included pacing, circling, and sleeping.

A buccal swab sample obtained when the puppy was 7 weeks old was submitted to the University of Missouri (Columbia, MO, USA) for DNA testing which confirmed that the puppy was homozygous for a missense mutation [c.152T>G, p.Met51Arg (NC_006618.3: dog chromosome 36: g. 19078954A>C)] in *ATF2* known to cause NEwS (1). Since there are no effective treatments for and this puppy was not able to feed herself, the owner requested euthanasia and offered to donate this 64-day-old puppy for the research. Investigations were conducted under the approval of the Institutional Animal Care and Use Committee and Animal and Human biology Research Ethics Committee of Nippon Veterinary and Life Science University (accession nos. 2019-K1, S2019-K1).

The body condition was thin. Abnormalities detected by neurological examination included cerebellar ataxia with hypermetria (Supplementary Video 2), moderately decreased postural reactions in all four limbs and an absent bilateral menace response. The examinations of the cranial nerves and the withdrawal reflexes were unremarkable. No crossed extensor reflexes were observed in all limbs. The CBC was unremarkable. Serum chemistry revealed increased alkaline phosphatase, total cholesterol, creatine kinase, inorganic phosphorus, and potassium, and decreased blood urea nitrogen and creatinine (Details are provided in Supplementary Appendix 1). Urinalysis showed decreased urine specific gravity of 1.023. The electrocardiography was unremarkable.

**Figure 1:**
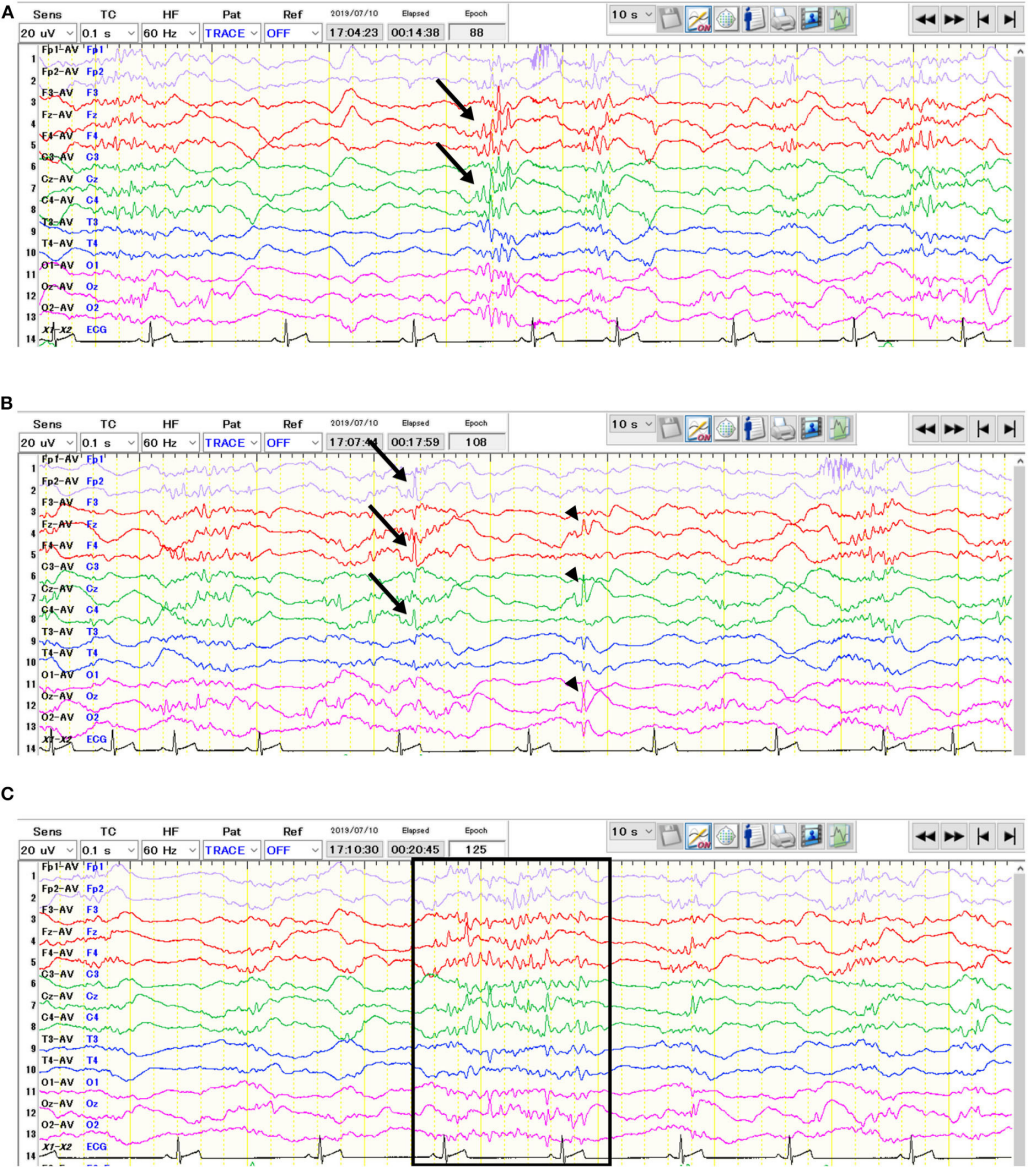
Interictal electroencephalogram of the 64-days-old puppy with neonatal encephalopathy with seizures. The average potential reference method was used. Polyspikes (**A**, arrows) and isolated spikes (**B**, arrows & arrowheads) were observed. In addition, alpha-band rhythms consisted of spikes and sharp waves (**C**, surrounded by the rectangle) were also seen.

The EEG was obtained under sedation using dexmedetomidine (20 μg/kg IV). Three additional administrations of dexmedetomidine (10 μg/kg IV) were given since the dog was not adequately sedated. Electrodes were placed as previously described (2). The EEG recording was made with a digital EEG system (Neurofax EEG-1200, Nihon Kohden, Tokyo, Japan) using averaged referential and bipolar montages (Fp1, Fp2, F3, Fz, F4, C3, Cz, C4, T3, T4, O1, Oz, O2). Other recording conditions were described in Supplementary Appendix 1. The visual EEG evaluation showed high-voltage slow frequency waves as background activity. Frequent spikes, polyspikes, and alpha-band rhythms consisted of spikes and sharp waves often predominantly originated from the central frontal (Fz) and/or parietal (Cz) region(s), with or without adjacent regions (Figure 1). Furthermore, isolated spikes were also seen at the right frontal pole (Fp2), frontal (F4), and parietal (C4) regions, or at the midline regions (Fz, Cz, and Oz) (Figure 1B). Photic stimulation did not increase the frequency of theses paroxysmal discharges. After the EEG recording, atipamezole (250 μg/kg IV) was administrated.

**Figure 2:**
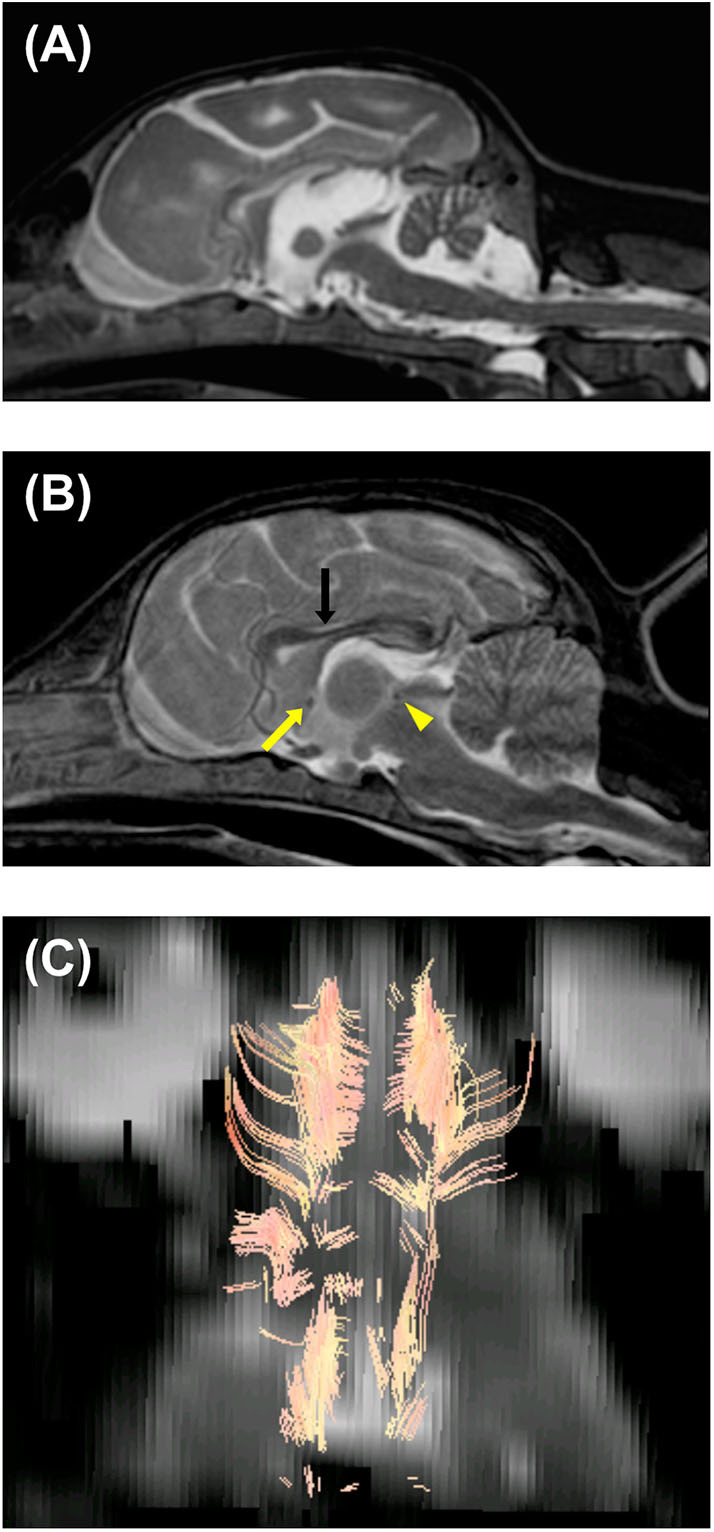
Magnetic resonance T2-weighted imaging of the brain in the median sagittal plane of the patient (64-days-old) **(A)** and a normal beagle dog (73-days-old) as a similar-age reference **(B)**. **(A)** Dilation of the third and fourth ventricles, presumptive dilatation of the pineal recess or a quadrigeminal cyst, and diffuse hypoplasia of corpus callosum, rostral commissure, caudal commissure, and the ventral part of the cerebellar vermis (lingula, nodulus, and uvula of the cerebellum) are seen. The reduced size of the brainstem and cerebellum and thinning of the interthalamic adhesion are also seen. **(B)** Unlike the patient, corpus callosum (black arrow), rostral commissure (yellow arrow), and caudal commissure (yellow arrowhead) are clearly recognized in a normal beagle dog (73-days-old). **(C)** Diffusion tensor imaging (DTI) tractography of the patient's brain. Regions of interest were placed to visualize corpus callosum. There seemed to be no visible commissural fiber across both hemispheres. This finding demonstrated hypoplasia of corpus callosum, although there is a possibility that DTI tractography may not accurately represent the white matter structure.

**Figure 3:**
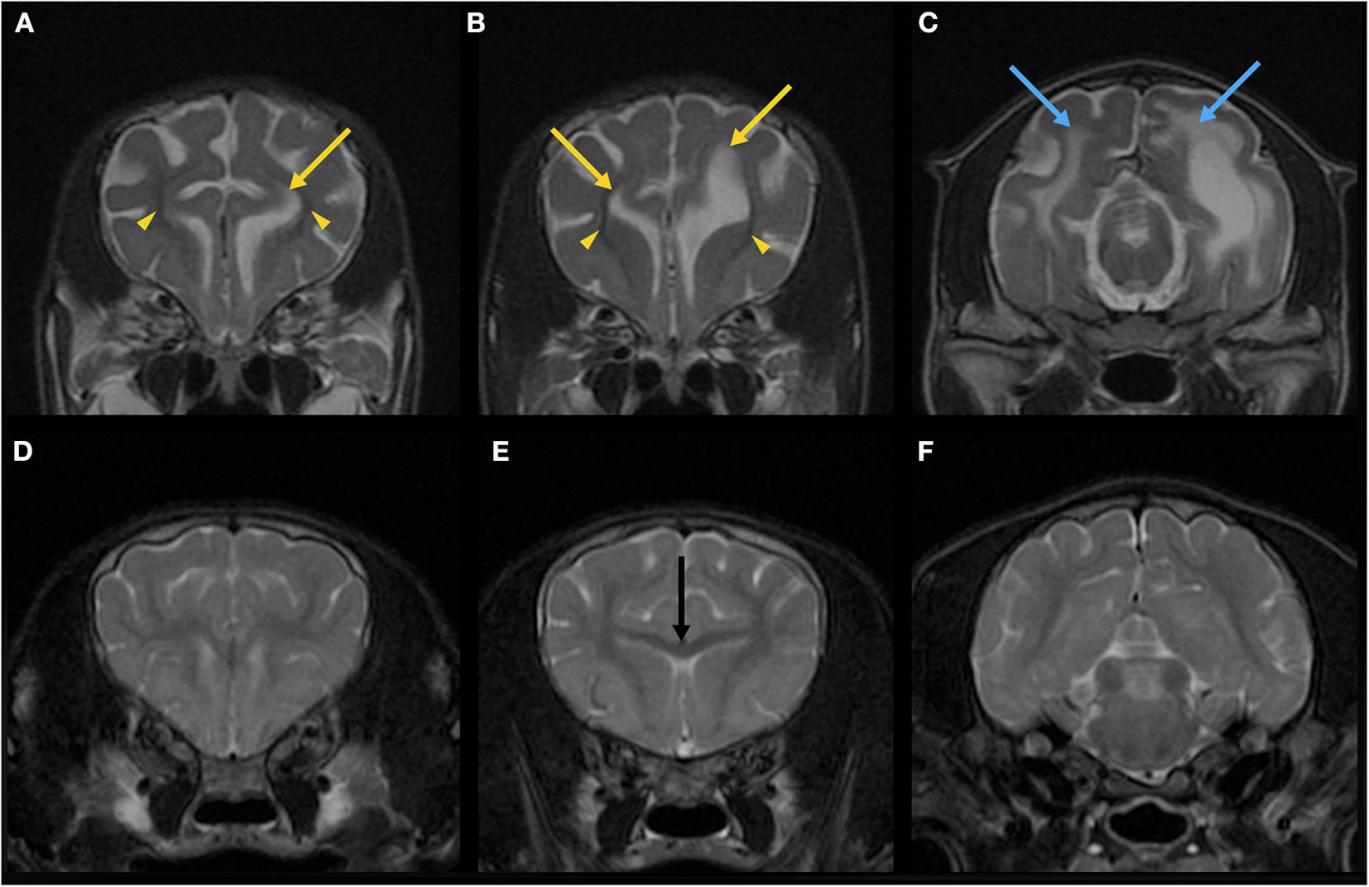
Magnetic resonance T2-weighted imaging (T2WI) of the brain in the transverse plane of the patient (64-days-old) **(A–C)** and a normal beagle dog (73-days-old) as a similar-age reference **(D–F)**. On the T2WI at the levels of the frontal lobe of the patient **(A,B)**, the lateral ventricles seemed to have upturned pointed corners (yellow arrows). Marked hypoplasia of subcortical white matter is also seen (yellow arrowheads). The brain sulci are clearly visible due to the suspected developmental abnormality. On the T2WI at the level of the occipital lobe of the patient **(C)**, there are T2-hyperintense lesions (blue arrows) in the right and left white matter in addition to the dilated left lateral ventricle. Corpus callosum (**E**, black arrow) is clearly seen in a 73-days-old normal beagle dog **(E)**, but not in the patient **(B)**.

Immediately after the EEG, MRI of the brain and the spinal cord was obtained using a 3.0-Tesla unit (Signa HDtx 3.0T, GE Healthcare, Tokyo, Japan) under general anesthesia with propofol induction and isoflurane maintenance. Details of sequences obtained were described in Supplementary Appendix 1. Since the patient was a 64-days-old puppy and the brain should be immature, conventional MRI previously obtained from a 73-days-old normal Beagle dog as a part of another study was used as a reference (Figures 2B, 3D–F). The MRI showed the reduced size of the whole brain including the cerebellum and brainstem, dilation of the ventricular system, presumptive dilatation of the pineal recess or an arachnoid diverticulum, subcortical white matter hypoplasia, and diffuse hypoplasia of corpus callosum, rostral commissure, caudal commissure, and the ventral part of the cerebellar vermis (lingula, nodulus, and uvula of the cerebellum) (Figures 2A, 3A,B). Those findings were not recognized in MRI of a 73-days-old normal Beagle dog used as a reference (Figures 2B, 3D,E). The fiber tractography based on diffusion tensor imaging (DTI) was generated using Functool which is an internal software of the MRI unit (GE Healthcare) supported the diffuse hypoplasia of the corpus callosum (Figure 2C). Fractional anisotropy (FA) values of the anterior internal capsule and the posterior internal capsule, which were calculated by DTI, were ~0.43 and 0.415, respectively. Those values were lower and higher, respectively, when compared with previously reported FA values of normal 9-week-old dogs (approximately 0.475 for the anterior internal capsule and approximately 0.34 for the posterior internal capsule) (3). In addition, at the level of the frontal lobe, the T2-weighted imaging (T2WI) of the lateral ventricles seemed to show upturned pointed corners (Figures 3A,B). This finding is often seen in canine patients with corpus callosum aplasia (4). Furthermore, marked T2WI and fluid-attenuated inversion recovery (FLAIR) hyperintensity and T1-weighted imaging (T1WI) hypointensity, not enhanced with gadodiamide, was observed bilaterally in the occipital white matter and more evident in the left hemisphere (Figure 3C). This region showed an increased apparent diffusion coefficient (ADC) value (1.8 × 10^−3^ mm^2^/s), which was calculated by diffusion-weighted imaging, and a decreased FA value (0.15) when compared with the previously reported values from normal 9-week-old dogs [ADC, approximately 0.9 × 10^−3^ mm^2^/s (the subcortical white matter); FA, approximately 0.475 and 0.34 (the anterior internal capsule and the posterior internal capsule, respectively)] (3, 5). Single-voxel magnetic resonance spectroscopy (MRS) was successfully obtained only at the fronto-parietal lobes. Lactate and lipid peaks were detected (Supplementary Figure 1), which are not typically seen in normal brain tissue, although lactate is known to be present in the cerebrospinal fluid (CSF) (6–9).

Whole-body computed tomography was obtained with an 80-slice CT scanner (Aquilion PRIME TSX-303A, Canon Medical Systems, Tochigi, Japan). No obvious abnormalities were found. CSF analysis was not available due to the contamination with blood when obtaining by cisternal puncture. Immediately after the above examinations, the puppy was euthanized, using propofol (30 mg/kg IV) followed by potassium chloride (2 mEq/kg IV).

**Figure 4:**
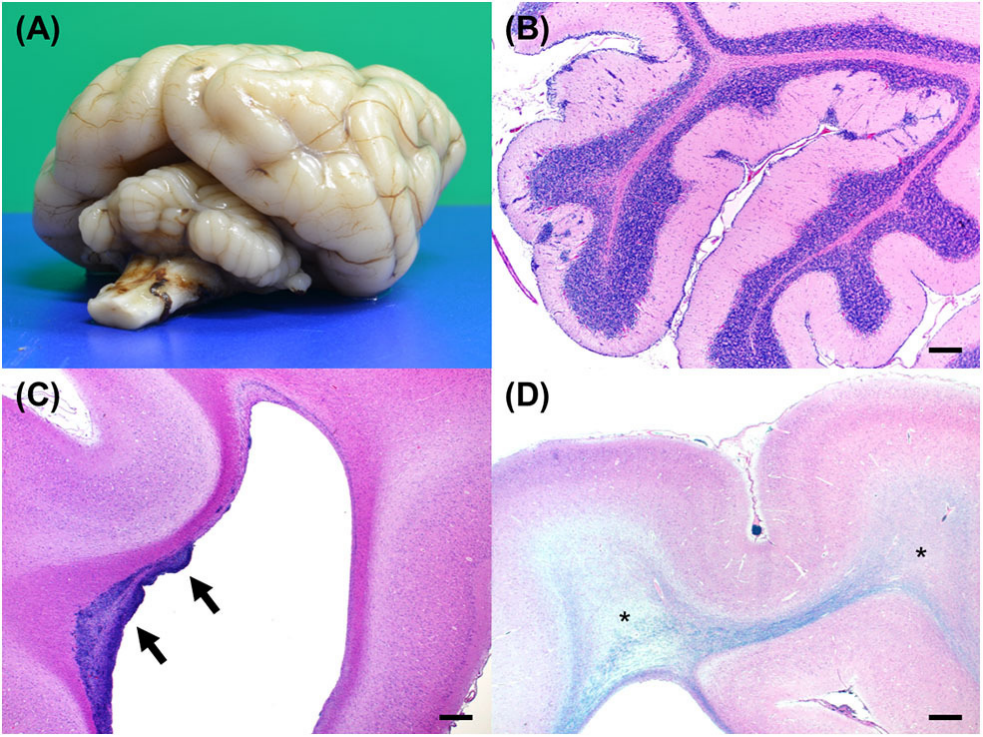
Gross and histopathological necropsy findings. **(A)** A diagonal view of the fixed brain shows hypoplasia of the cerebellum. The cerebellar vermis is markedly small. **(B)** An HE-stained section of the cerebellum shows clusters of ectopic cells in the cerebellar cortex. Bar = 500 μm. **(C)** An HE-stained section of the cerebrum shows clusters of cells in the wall of lateral ventricle in the frontal lobe (arrows). Bar = 500 μm. **(D)** Luxol fast blue and HE-stained section of the cerebrum shows rarefaction of the white matter with hypomyelination (asterisks). Bar = 500 μm.

After euthanasia, a complete necropsy was performed. Gross examination showed hypoplasia of the ventral part of the cerebellar vermis (Figure 4A) and dilation of the left lateral ventricle. Histopathological examination revealed no significant findings beyond the central nervous system. In the cerebellar cortex, some irregularities in the layer structure were found. At the same site, molecular layers and internal granule layers were mixed, and Purkinje cells were found ectopically. In addition, the molecular layer showed scattered nests of granular cells and Purkinje cells. An external granular layer was also observed. These findings seen in the cerebellar cortex were consistent with the previously reported findings in NEwS (Figure 4B) (1). However, the abnormal-shaped part of the cerebellum did not show the abnormal layer structure while the normal-shaped part showed the histopathological changes.

Mild sporadic neuronal atrophy was found in the cerebrum. Undifferentiated cells were multifocally or diffusely distributed in the subventricular zone of the frontal lobe (Figure 4C). Adjacent areas contained sporadic accumulations of immature neurons. Furthermore, white matter rarefaction with hypomyelination was found at the level of the occipital lobe (Figure 4D), which corresponded to the lesion with T2- and FLAIR hyperintensity, T1-hypointensity, no contrast enhancement, increased ADC value, and decreased FA value in MRI (Figure 3C). Although less apparent than in the occipital lobe, rarefaction with hypomyelination was also identified in the frontal lobe histopathologically.

The siblings were genotyped for the causal variant in *ATF2* (c.152T>G) by PCR and Sanger sequencing (Supplementary Appendix 1). Three of the siblings were wildtype and three were heterozygous for this variant. None of these full-siblings showed seizures, ataxia or other neurological abnormalities.

## Discussion

In the current report, we described MRI findings and previously unreported histopathological findings in a puppy with NEwS. Of note were neurodevelopmental changes, including reduced whole-brain size, subcortical white matter hypoplasia, white matter rarefaction with hypomyelination, and diffuse hypoplasia of the corpus callosum, rostral commissure, caudal commissure, and the ventral part of the cerebellar vermis.

In the present case, the EEG contained polyspikes and alpha-band rhythms consisted of spikes and sharp waves in addition to isolated spikes that have been previously reported (1). While the previous report used a simple five-lead montage with each frontal and occipital region referenced to the vertex (F3, F4, O1, O2, Cz), we used 13 channels (Fp1, Fp2, F3, Fz, F4, C3, Cz, C4, T3, T4, O1, Oz, O2) with averaged referential and bipolar montages, which enabled us to evaluate electrophysiological conditions in more detail. This 9-week-old puppy had generalized epileptiform discharges such as spikes and polyspikes which predominantly originated from the central frontal (Fz) and/or parietal (Cz) region(s) as well as isolated spikes which were seen at the right frontal pole (Fp2), frontal (F4), and parietal (C4) regions, or at the midline regions (Fz, Cz, and Oz) (Figure 1). In contrast, a 4-week-old puppy in the previous study showed spikes and slow waves most prominent in the occipital lobes. This may reflect the disease progression and/or variations in disease expression.

Here, we present the MRI findings and previously unrecognized histopathological findings in a dog with NEwS. First, an abnormal shape of the cerebellum was revealed in the MRI (Figure 2A). This finding corresponded to the reduced size of the cerebellum as reported in the previous study (1). Interestingly, the cerebellar cortical dysplasia was not found in the abnormally shaped region but instead in the normally shaped regions. A previous study of ATF2-deficient mice (10) has demonstrated that ATF2 is essential for correct neurological development and neuronal migration.

Second, the MRI showed that the brainstem was markedly reduced in size (Figure 2A). This finding has not been reported in the previous report, however, the reduced size of brainstem as well as the cerebellum has reported in neuron-specific ATF2 deleted mice (11). This finding may demonstrate the importance of ATF2 in the growth process of the rhombencephalon in dogs.

Third, abnormalities of the cerebral white matter were found. MRI and/or histopathology demonstrated subcortical white matter hypoplasia (Figures 3A,B), white matter rarefaction with hypomyelination (Figures 3C, 4D), and diffuse hypoplasia of the corpus callosum, rostral commissure, and caudal commissure (Figure 2). Interestingly, these lesions were not included in an earlier description of dogs with NEwS. Although the progressive myelination of the cerebral white matter is a hallmark of postnatal brain maturation, the full length of the corpus callosum has been observed at 8 weeks in T2WI or at 6 weeks in T1WI in canine brains (5). A limitation is that we did not have completely age- and breed-matched (9-week-old) controls for MRI and histopathological analyses. However, MRI of a similar-aged normal beagle dog (73-days-old) did not show those findings seen in the patient with NEwS (Figures 2B, 3D–F), and DTI tractography demonstrated hypoplasia of white matter structure in this case (Figure 2C). Furthermore, FA values of the anterior internal capsule and the posterior internal capsule were measured. Those values were lower and higher, respectively, than those reported previously in normal 9-weeks-old dogs (3). Interestingly, a similar finding was reported in a 9-weeks-old dog with Krabbe disease in the same study cited above (3). As an additional possible limitation, the DTI measurement and tractography are known to be influenced by the presence of edema. Therefore, it is possible that the DTI measurement and tractography may not accurately represent the white matter structure and condition. However, this finding may be associated with rarefaction with hypomyelination seen in the frontal lobe white matter though it was less significant than that seen in the occipital lobe, which was confirmed by histopathology. In the progression of myelination in the subcortical white matter, a higher organization of the myelinated fibers and the minimum amount of fluid in canine brain were observed by 8 weeks histologically (5). As the canine brain matures, the subcortical white matter is T2-hyperintense to gray matter during the juvenile phase (at 1–4 weeks) in MRI (5). However, T2-hyperintensity of the subcortical white matter is not seen at 6 weeks (transitional phase) and later (5). Those findings seen in this dog may be consistent with a white matter developmental abnormality, and may have partially contributed to the enlarged sulci seen in the brain.

Additionally, single-voxel MRS of the fronto-parietal lobes detected lactate and lipid peaks (Supplementary Figure 1), which are generally unclear in the normal brain tissue. Lactate is a byproduct of anaerobic metabolism and the lactate peak (1.3 ppm) is especially known to be an indication of nonoxidative metabolism (12). The lipid peak (0.9 ppm) indicates the presence of cellular membrane breakdown or necrosis though voxel contamination of fatty tissues such as scalp, subcutaneous tissue, and diploic space (12). It has also been reported that lactate concentration was increased by seizure in humans and animal models (13–16). However, due to the enlarged ventricles, the voxel of interest included the ventricles. Some studies found lactate in the spectra of CSF from individuals with or without disease conditions (6–8). One study also proposed that the detection of lactate in spectra obtained from voxels including ventricular CSF has no pathophysiological significance and should be interpreted with caution (9). Therefore, the peak of lactate found in the affected puppy may be due to the contamination of CSF, and it is unclear if the peak of lactate has pathophysiological significance in this case. On the other hand, there is a possibility that the lipid peak seen in this puppy might be indicative of underlying abnormal brain conditions.

Finally, changes in the subventricular zone were consistent with a neuronal migration abnormality (Figure 4C), which has never been reported in dogs with NEwS. ATF2 was suggested to be crucial for promoting brain progenitor cell survival and differentiation (17). ATF2 dysfunction resulting from the missense mutation in *ATF2* may be responsible for the neuropathology in the affected puppy. As with ATF2 knockout mice (10), enlarged ventricles were also found in this case. Furthermore, other findings seen in this case such as ectopic Purkinje and granular cells and intermixed molecular and internal granule layers were consistent with those reported previously (1), which were considered as abnormal findings. Although an external granular layer was found in this case, it has been reported that a vestige of the external granular layer may remain at ~10 weeks (18). Therefore, since this case was a 64-days-old puppy, an external granular layer found in this case may not be an abnormal finding.

Chondrodysplasia reported in ATF2 deficient mice (10) was not detected in this puppy. Although ATF2 null mice were also reported to show severe respiratory distress followed by neonatal death with lungs filled with meconium (19), there were no remarkable clinical respiratory signs, computed tomography findings, or pathological changes in the lung in the affected puppy. These phenotypic features seen in mice may be due to the null mutation and/or differences between species.

A limitation of this study is that completely age- and breed-matched controls were not available for comparison especially for analyses of MRI, including conventional sequences, DTI, and MRS, and histopathological findings. Furthermore, various examinations, such as MRI and EEG, were performed only once, and there was no CSF analysis.

In conclusion, we characterized the MRI findings and the more detailed EEG and histopathological findings of NEwS in a Standard Poodle puppy with a known *ATF2* homozygous missense mutation. Our findings may offer new insights into function of ATF2 associated with neurodevelopment.

## Data Availability Statement

All datasets generated for this study are included in the article/Supplementary Material.

## Ethics Statement

The animal study was reviewed and approved by the Institutional Animal Care and Use Committee and Animal and Human biology Research Ethics Committee of Nippon Veterinary and Life Science University (accession nos. 2019-K1, S2019-K1). Written informed consent was obtained from the owner for the participation of the animals in this study.

## Author Contributions

YY and GJ: conception and design. YY, JC, KK, and RA: acquisition of data. YY: drafting the article. YY, DH, JC, KK, GJ, and KU: analysis and interpretation of data. All authors: revising article for intellectual content and final approval of the completed article.

## Conflict of Interest

GJ heads a laboratory that conducts fee-for-service DNA testing for NEwS. The remaining authors declare that the research was conducted in the absence of any commercial or financial relationships that could be construed as a potential conflict of interest.

## Supplementary Material

Supplementary Figure 1 Spectrum of single-voxel proton magnetic resonance spectroscopy (H1-MRS) of the fronto-parietal lobe using short echo time sequence. The lactate peak (1.3 ppm) and lipid peak (0.9 ppm) are seen. Cho, choline; Cr, creatine; NAA, N-acetylaspartate; mI, myo-inositol.

Supplementary Table 1 Pathogenicity prediction of the amino acid change from methionine (Met) to arginine (Arg) at residue 51 of ATF2 causing NEwS.

Supplementary Video 1 The Standard Poodle puppy having generalized seizure due to neonatal encephalopathy with seizures (NEwS).

Supplementary Video 2 The Standard Poodle puppy with NEwS displaying ataxia.

Supplementary Appendix 1 Serum chemistry, Electroencephalography recording conditions, Details of MRI sequences, Histopathological Methods, DNA Sampling, DNA isolation, and genotyping of the siblings, and Pathogenicity prediction of the *ATF2* variant.
