# Supplementary material for: Magnetic Resonance Imaging and Histopathologic Findings From a Standard Poodle With Neonatal Encephalopathy With Seizures
Source: Front Vet Sci. 2020 Nov 10;7:578936. doi: 10.3389/fvets.2020.578936 (PMC7683776; doi:10.3389/fvets.2020.578936)
Supplement: Supplementary Figure 1 — Spectrum of single-voxel proton magnetic resonance spectroscopy (H1-MRS) of the fronto-parietal lobe using short echo time sequence. The lactate peak (1.3 ppm) and lipid peak (0.9 ppm) are seen. Cho, choline; Cr, creatine; NAA, N-acetylaspartate; mI, myo-inositol. [file Data_Sheet_2.PDF]

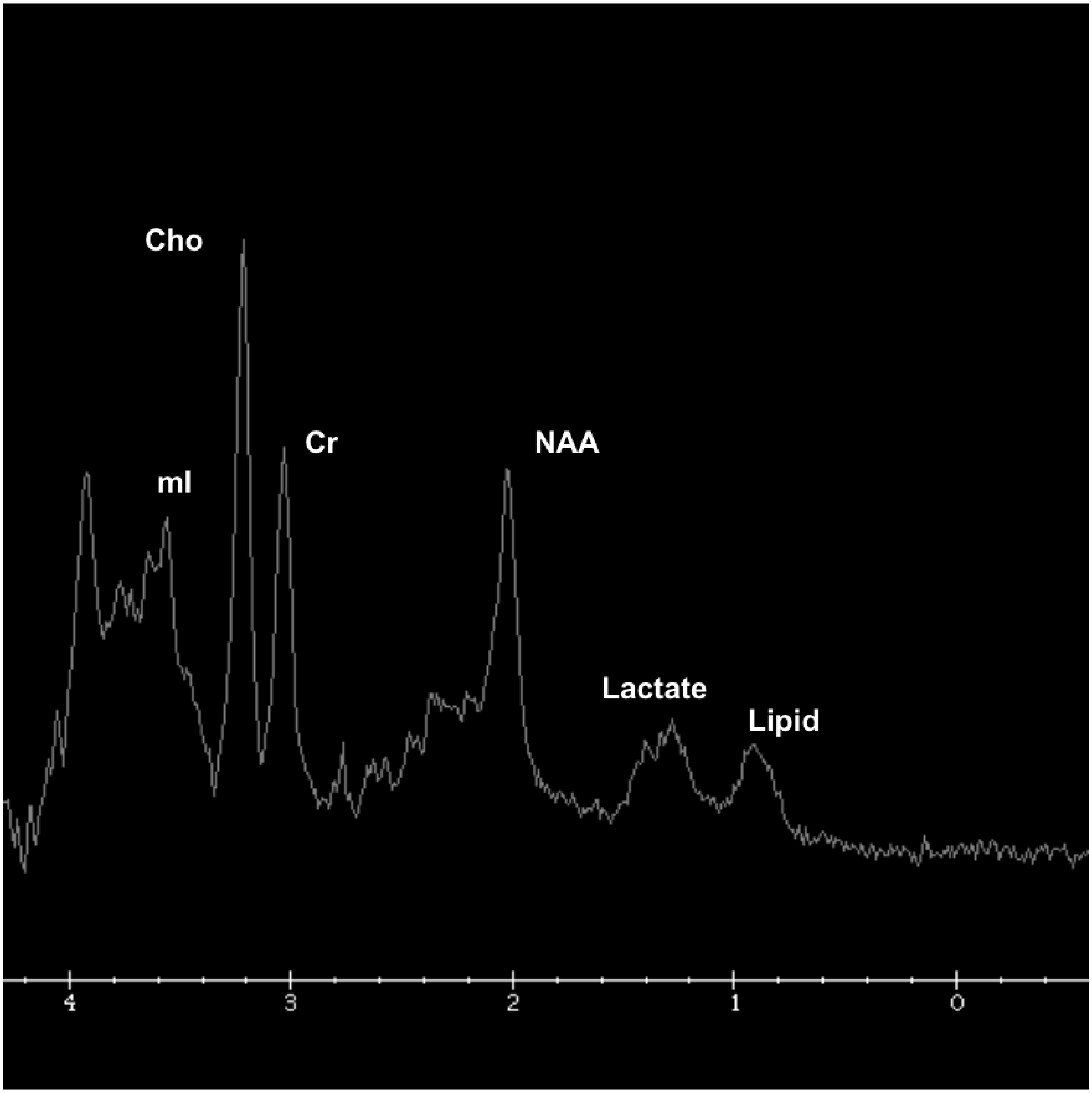

FIGURE S1. Spectrum of single-voxel proton magnetic resonance spectroscopy (H1-MRS) of the fronto-parietal lobes using short echo time sequence. The lactate peak (1.3 ppm) and lipid peak (0.9 ppm) are seen. Cho: choline, Cr: creatine, NAA: N-acetylaspartate, mI: myo-inositol.
