# Supplementary material for: Magnetic Resonance Imaging and Histopathologic Findings From a Standard Poodle With Neonatal Encephalopathy With Seizures
Source: Front Vet Sci. 2020 Nov 10;7:578936. doi: 10.3389/fvets.2020.578936 (PMC7683776; doi:10.3389/fvets.2020.578936)
Supplement: Supplementary Table 1 — Pathogenicity prediction of the amino acid change from methionine (Met) to arginine (Arg) at residue 51 of ATF2 causing NEwS. [file Data_Sheet_3.PDF]

Table S1. Pathogenicity prediction of the amino acid change from methionine (Met) to arginine (Arg) at residue 51 of ATF2 causing NEwS.

In silico tools	Pathogenicity prediction	Pathogenicity scores	Pathogenicity threshold	Predicted molecular mechanism (*P*-value ≤ -0.05)
Provean	Deleterious	-5.035	≤ -2.5	
PolyPhen-2	Probably Damaging	0.991 (HumVar)	Probably damaging: 0.801–1.000	
MutPred2	Deleterious	0.846	> 0.5	Loss of SUMOylation at K48 (*P* = 0.04), Altered metal binding (*P* = 0.04)
SNPs&GO	Disease	0.743	> 0.5	
