# Supplementary material for: Magnetic Resonance Imaging and Histopathologic Findings From a Standard Poodle With Neonatal Encephalopathy With Seizures
Source: Front Vet Sci. 2020 Nov 10;7:578936. doi: 10.3389/fvets.2020.578936 (PMC7683776; doi:10.3389/fvets.2020.578936)
Supplement: Supplementary Appendix 1 — Serum chemistry, Electroencephalography recording conditions, Details of MRI sequences, Histopathological Methods, DNA Sampling, DNA isolation, and genotyping of the siblings, and Pathogenicity prediction of the ATF2 variant. [file Data_Sheet_1.PDF]

Appendix S1

Serum chemistry

Serum chemistry revealed increased alkaline phosphatase (1105 U/L; ref. 0–254), total cholesterol (396 mg/dL; ref. 111–312), creatine kinase (604 U/L; ref. 50–170), inorganic phosphorus (7.3 mg/dL; ref. 1.9–5.0), potassium (6.2 mEq/L; ref. 3.8–5.0), decreased blood urea nitrogen (6.7 mg/dL; ref. 9.2–29.2) and creatinine (0.30 mg/dL; ref. 0.40–14.0).

Electroencephalography recording conditions

Detailed recording conditions were as follows: sampling frequency = 1000 Hz; high frequency filter = 60 Hz; time constant = 0.1; sensitivity = 20 μV/mm; AC cut-off notch filter = ON; and tracing speed = 10 s/view. The EEG was recorded for 30 minutes.

Details of MRI sequences

The following MRI sequences were obtained for brain MRI: transverse T2-weighted imaging (T2WI), fluid-attenuated inversion recovery (FLAIR), T1-weighted imaging (T1WI), dorsal FLAIR, three-dimensional (3D) T2 Cube, 3D T1WI, diffusion-weighted imaging (DWI), diffusion tensor imaging (DTI), and single-voxel magnetic resonance spectroscopy (MRS). These included sequences comparable to a veterinary epilepsy-specific MRI protocol (1). DWI was obtained using spin echo-echo planar imaging periodically rotated overlapping parallel lines with enhanced reconstruction (PROPELLER), and parameters for DWI were as follows: echo time (TE), 72.2 ms; repetition time (TR), 8000ms; field of view (FOV), 15 × 15; slice thickness, 3.0 mm; interslice gap, 0.5 mm; matrix, 128 × 128; number of excitations (NEX), 1; motion probing gradient, 3 axes; b value = 1000 s/mm2. DTI was obtained using spin echo-echo planar imaging (SE-EPI), and parameters for DTI were as follows: TE, 97 ms; TR, 8000 ms; FOV: 15 × 15; slice thickness, 2.4 mm; interslice gap, 0.5 mm; matrix, 128 × 128; NEX, 2; motion probing gradient, 15 axes; b value = 1000 s/mm2. Single-voxel MRS was performed using a point-resolved spectroscopic localization sequence (PRESS) with the following parameters using spin echo-echo planar imaging: TE, 35 ms; TR, 2000 ms; NEX, 8. The voxel of interest (VOI) for single-voxel MRS was placed at the fronto-parietal lobes including both hemispheres (20.1 mm × 20.3 mm × 18.2 mm), and the cerebellum (10.2 mm × 10.0 mm × 11.5 mm). Due to the enlarged ventricles, the VOI at the fronto-parietal lobes included the ventricular CSF in addition to the brain tissue. Subsequently, after IV administration of gadodiamide (0.05 mmol/kg), transverse T1WI and 3D T1WI were repeated. Furthermore, dorsal T2WI of the spinal cord was obtained.

Histopathological Methods

Tissues were fixed in 10% neutral-buffered formalin and embedded in paraffin wax. Four μm-thick sections were stained with hematoxylin and eosin (HE) and Luxol fast blue.

DNA Sampling, DNA isolation, and genotyping of the siblings

Blood for CBC and serum chemistry was collected from the affected puppy by jugular venipuncture. The remaining blood in ethylenediaminetetraacetic acid tubes was used for the genetic testing. DNA was extracted using QIAamp DNA mini kit (Qiagen, Hilden, Germany) according to the manufacturer's instructions. PCR was performed using AmpliTaq Gold 360 Master Mix (Applied Biosystems, Foster City, CA, USA) in a 25 µl reaction volume using

forward (5’-TGGTGGTTTTACATGTTGTGG-3’) and reverse (5’-AATGGTCAGAAGGAAACATGG-3’) primers. After the initial denaturation at 95 °C for 10 min, the following steps were taken for 40 cycles: denaturation at 95 °C for 30 sec, annealing at 59 °C for 30 sec, and extension at 72 °C for 1 min. A final extension at 72 °C for 7 min was carried out. PCR products were purified using Wizard SV Gel and PCR Clean-Up System (Promega, Madison, WI, USA) and then Sanger sequenced bi-directionally on an Applied Biosystems 3730xl DNA Analyzer (Applied Biosystems). Buccal swab samples were also collected from the surviving siblings and genotyped.

Pathogenicity prediction of the *ATF2* variant

The missense variant was analyzed *in silico* using PROVEAN (http://provean.jcvi.org/index.php) (2), PolyPhen-2 (http://genetics.bwh.harvard.edu/pph2/) (3), MutPred2 (http://mutpred.mutdb.org/) (4), and SNPs&GO (http://snps.biofold.org/snps-and-go/snps-and-go.html) (5). The pathogenicity of an amino acid change from methionine (Met) to arginine (Arg) at residue 51 of ATF2 was predicted as Deleterious, Probably Damaging, or Disease. Detailed results were summarized in **Table S1**.

REFERENCES

1. Rusbridge C, Long S, Jovanovik J, Milne M, Berendt M, Bhatti SFM, et al. International Veterinary Epilepsy Task Force recommendations for a veterinary epilepsy-specific MRI protocol. *BMC Vet Res* (2015) **11**:194. doi:10.1186/s12917-015-0466-x

2. Choi Y, Chan AP. PROVEAN web server: a tool to predict the functional effect of amino acid substitutions and indels. *Bioinformatics* (2015) **31**:2745–2747. doi:10.1093/bioinformatics/btv195

3. Adzhubei I, Jordan DM, Sunyaev SR. Predicting Functional Effect of Human Missense Mutations Using PolyPhen-2. *Current Protocols in Human Genetics* (2013) **76**:7.20.1-7.20.41. doi:10.1002/0471142905.hg0720s76

4. Pejaver V, Urresti J, Lugo-Martinez J, Pagel KA, Lin GN, Nam H-J, et al. MutPred2: inferring the molecular and phenotypic impact of amino acid variants. Bioinformatics (2017). doi:10.1101/134981

5. Capriotti E, Calabrese R, Fariselli P, Martelli P, Altman RB, Casadio R. WS-SNPs&GO: a web server for predicting the deleterious effect of human protein variants using functional annotation. *BMC Genomics* (2013) **14**:S6. doi:10.1186/1471-2164-14-S3-S6
